# Ankyrin G Membrane Partners Drive the Establishment and Maintenance of the Axon Initial Segment

**DOI:** 10.3389/fncel.2017.00006

**Published:** 2017-01-26

**Authors:** Christophe Leterrier, Nadine Clerc, Fanny Rueda-Boroni, Audrey Montersino, Bénédicte Dargent, Francis Castets

**Affiliations:** CNRS, CRN2M, Aix Marseille UniversityMarseille, France

**Keywords:** ankyrin G, axon initial segment, neurofascin-186, voltage gated sodium channel, organotypic slices, cultured hippocampal neurons

## Abstract

The axon initial segment (AIS) is a highly specialized neuronal compartment that plays a key role in neuronal development and excitability. It concentrates multiple membrane proteins such as ion channels and cell adhesion molecules (CAMs) that are recruited to the AIS by the scaffold protein ankyrin G (ankG). The crucial function of ankG in the anchoring of AIS membrane components is well established, but a reciprocal role of membrane partners in ankG targeting and stabilization remained elusive. In rat cultured hippocampal neurons and cortical organotypic slices, we found that shRNA-mediated knockdown of ankG membrane partners (voltage-gated sodium channels (Nav) or neurofascin-186) led to a decrease of ankG concentration and perturbed the AIS formation and maintenance. These effects were rescued by expressing a recombinant AIS-targeted Nav or by a minimal construct containing the ankyrin-binding domain of Nav1.2 and a membrane anchor (mABD). Moreover, overexpressing mABD in mature neurons led to ankG mislocalization. Altogether, these results demonstrate that a tight and precocious association of ankG with its membrane partners is a key step for the establishment and maintenance of the AIS.

## Introduction

Neurons exhibit an axonal/dendritic polarity that allows the directional propagation of signals throughout the nervous system. A major player in the maintenance of this polarity is the axon initial segment (AIS), a specialized domain located within the first 50 μm of the axon. The AIS isolates the axon from the cell body and regulates protein exchange between the axonal and somatodendritic compartments (Rasband, 2010; Leterrier, 2016). Besides this important role in neuronal polarity, the AIS is also the site of generation of action potentials (Clark et al., 2009; Kole and Stuart, 2012). At the molecular level, the AIS is organized by ankyrin G (ankG), a specialized scaffolding protein that directly binds to the submembrane cytoskeletal lattice of ßIV-spectrin (ßIVsp) and actin. The amino-terminus of ankG, called the membrane-binding domain (MBD), is apposed to the inner face of the plasma membrane and directly interacts with transmembrane proteins such as voltage-gated sodium channels (Nav), potassium channels Kv7.2/3 (KCNQ2/KCNQ3), and the cell adhesion molecules (CAMs) NrCAM and Neurofascin-186 (Nfasc186; Xu et al., 2013; Leterrier et al., 2015). AnkG is also associated with the microtubule cytoskeleton by a direct interaction with End-Binding proteins (EB1 and EB3; Leterrier et al., 2011b; Fréal et al., 2016).

AnkG is considered to be the master organizer of the AIS (Rasband, 2010). It is the earliest component addressed to the AIS and is responsible for the subsequent recruitment of most AIS-enriched proteins (Jenkins and Bennett, 2001; Hedstrom et al., 2008; Galiano et al., 2012). AnkG depletion not only impairs the accumulation of other AIS components, but also causes a progressive loss of neuronal polarity (Hedstrom et al., 2007; Sobotzik et al., 2009). Precisely how and where ankG interacts with its membrane partners during AIS formation and maintenance is still elusive. The “diffusion and trapping” model proposes that an existing ankG scaffold immobilizes membrane proteins at the AIS (Brachet et al., 2010; Xu and Cooper, 2015). Alternatively, recent data suggest that a preformed complex of ankG and Nav is transported to the AIS (Barry et al., 2014) and that Nav can be directly inserted at the AIS (Akin et al., 2015). Moreover, several results suggest an interplay between ankG and its membrane partners: the establishment of the AIS is impaired in motor neurons depleted for Nav (Xu and Shrager, 2005) and Nfasc186 elimination in Purkinje cells of adult mice results in progressive AIS disassembly (Zonta et al., 2011). In addition, we have shown that perturbing the ankG/Nav interaction by inhibiting the protein kinase CK2 downregulates Nav accumulation at the AIS and subsequently decreases ankG concentration (Bréchet et al., 2008; Brachet et al., 2010). To reveal and characterize the interplay between ankG and its membrane partners, we have examined the role of AIS membrane proteins (Nav and Nfasc186) in AIS assembly and maintenance. We performed shRNA-mediated silencing of Nav and Nfasc186 in cultured neurons during and after the AIS formation as well as in organotypic slices. In all conditions, knockdown of Nav and Nfasc-186 resulted in a cumulative impairment of the AIS stability. Rescue experiments with a full-length Nav1.6 or a minimal construct combining the ankG binding domain of Nav and a membrane targeting motif showed that anchoring of ankG to the plasma membrane via its partners is necessary for its targeting and stable assembly at the AIS. Finally, overexpression of the membrane-anchored ankG-binding construct induced a mislocalization of ankG, suggesting that interaction of ankG with its membrane partners occurs before the insertion of ankG into the AIS.

## Materials and Methods

### Antibodies and Plasmids

A rabbit anti-ßIV spectrin antibody (Eurogentec) was developed against amino acid residues 15–38 of the human sequence (XP 006723369). Mouse monoclonal antibodies to ankG (1:400, N106/36 NeuroMab), Nfasc186 (1:200, L11A/41 NeuroMab) and sodium channels (pan Nav; 1:100; Sigma-Aldrich), rabbit polyclonal antibodies to GFP (1:1000; A11122 ThermoFisher), and chicken polyclonal antibodies to map2 (1:10,000; Abcam) were used. Secondary goat antibodies conjugated to Alexa Fluor 488, 555, 647 (ThermoFisher) or DyLight 405 (Jackson ImmunoResearch Laboratories) were used at 1:400 dilutions.

The nucleotide sequences of rat Nav1.2 fragment coding from amino acid 1081–1203 (wild type [WT], mutated for E1111, mutated for S1112/1123/1124/1126 or mutated for both E and the four S) were obtained by PCR amplification (Expand High Fidelity Taq polymerase, Roche Molecular Biochemicals) on pKv2.1-Nav1.2 plasmids (Bréchet et al., 2008) and inserted into the pEGFP-F (Clontech) in the 5′ of EGFP sequence. The resulting sequence encodes a chimeric protein we called membranous Ankyrin Binding Domain (mABD; see Figures 6A,B). All constructs were verified by DNA sequencing (Beckman Coulter Genomics).

### Animals, Cultured Hippocampal Neurons and Organotypic Slice Culture

All experiments were carried out in accordance to the guidelines established by the European Animal Care and Use Committee (86/609/EEC) and was approved by the ethic committee of Marseille N°14 (agreement D13-055-8 from French ESR Ministry). For primary neuronal cell culture, pregnant female Wistar rats (Janvier labs) were sacrificed by decapitation and E18 embryos brains were quickly removed. Hippocampal neurons were then prepared according to the Banker-type culture protocol (Kaech and Banker, 2006) and either nucleofected before plating using an Amaxa rat nucleofector kit (Lonza) according to the manufacturer’s intrusions or transfected at 8 days *in vitro* (8 div) using Lipofectamine 2000 (ThermoFisher). Cortical slices (350 μm) were made from rat pups of 7 days sacrificed by decapitation. They were cultured for 7 days according to the protocol described by Stoppini et al. (1991) and received microinjections of lentivirus (0.1–0.2 μl; titer from 10^8^ to 10^10^ pfu/ml) at the first day in culture.

### Lentivus Vectors

The pFUGW plasmid (Lois et al., 2002) was modified to express farnesylated EGFP (from pEGFP-F, Clontech), tdTomato (from ptdTomato-N1, Clontech) or mABD (see above) rather than EGFP. On the 5′ of the ubiquitin-C promoter (PacI site) specific shRNA expression cassette (U6 promoter/T5/shRNA/T5/H1promoter from pFIV-H1/U6 siRNA vector, System Biosciences) was introduced by restriction. The sequence of shRNAs directed against ankG, Nav1 and Nfasc186 have been previously described (Hedstrom et al., 2007). Luciferase shRNA (shLuc 159 also known as SHC007 sigma) was validated as a control (Abad et al., 2010). Modified plasmids were used either directly by transfection of neuronal cells or to produce pseudotyped lentivirus according to Dull et al. (1998). Lentivirus liberated in cell culture medium was concentrated by ultracentrifugation, titrated and kept at −80° in PBS-1% glycerol. The lentivirus production was performed in the lentivector production facility/SFR BioSciences Gerland—Lyon Sud (UMS3444/US8).

### Immunocyto- and Immunohistochemistry

Transfected cells were processed for immunofluorescence 6 or 7 days post transfection. They were fixed 10 min in 4% paraformaldehyde, permeabilized and blocked with 0.1% of Triton X-100 and 0.22% gelatin in 0.1 M phosphate buffer for 30 min and then incubated for 1 h with primary antibodies in the blocking solution. Corresponding secondary antibodies conjugated to Alexa Fluor or DyLight fluorophores were incubated for 1 h. Coverslips were mounted in Fluor Save reagent (EMD). Free-floating organotypic slices kept on hydrophilized PTFE membranes (Millipore) were fixed for 30 min in 4% paraformaldehyde 7 days post infection. They were blocked and permeabilized overnight in PBS containing 0.5% of Triton X-100, 1% Normal Goat Serum (NGS) and 100 μg/ml of Bovine Serum Albumin (BSA). Then, slices were incubated for 4 h in PBS containing 0.25% of Triton X-100, 0.5% NGS and 50 μg/ml of BSA and for 1 h in the corresponding secondary antibodies conjugated to Alexa Fluor fluorophores. Finally, slices were counterstained for nuclei with Hoechst 33342 (1 μg/mL) and dry-mounted in ProLong Gold (Life Technologies).

### Images Acquisition and Analysis

For organotypic slices, image acquisition was performed on a Zeiss LSM780 (Zeiss, Jena, Germany) confocal microscope equipped with a 63× 1.4 N.A. oil immersion objective. Three-dimensional *z*-stacks were collected automatically as frame by frame sequential image series (80–120 optical slices). For cultured hippocampal neurons, image acquisition was performed on a Zeiss Axio Imager Z2 equipped with a 40× 1.4 N.A. oil immersion objective. For illustration purposes, image editing was performed using ImageJ software^1^ and was limited to Sigma Plus Filter, linear contrast enhancement and gamma adjustment. Quantification was performed using ImageJ. Regions of interest corresponding to proximal axons or dendrites were selected on ankG or GFP images using NeuronJ plugin. The AIS was automatically detected along the proximal axon tracing by a custom ImageJ script (Leterrier et al., 2015)^2^, which determines the point of the maximum intensity of the ankG labeling. The beginning and the end of the AIS were determined from either sides at 33% of the maximum intensity along the tracing (Grubb and Burrone, 2010). The intensity of ankG labeling was then measured along the defined AIS segment with a 2 pixel (0.325 μm) width for each neuron. The mean intensity along the AIS of the transfected neurons was normalized by dividing it by the mean intensity along the AIS of untransfected surrounding neurons in the same image, allowing to circumvent variations related to the immunolabeling. All intensities were corrected for background signal (taken as the mode of the whole image histogram). To determine the intensity in channels other than ankG, the AIS segment region was detected on ankG labeling, then reported to the other channel and the intensity was measured as described above. Results are expressed as mean ± SEM. AIS/dendrite ratio is the mean labeling intensity of the proximal axon divided by the mean labeling intensity of three dendrites. The polarity index (for ankG quantification in organotypic slices) is the ratio of the mean labeling intensities: (AIS − soma) divided by (AIS + soma). The width was measured perpendicularly to the axon at the point of maximum intensity of ankG along the AIS. Statistical analyses were performed using Prism 5 (Graphpad Software, La Jolla, CA, USA). Significances were tested using two-tailed unpaired *t* tests (two conditions) or one-way ANOVA followed by Kruskal-Wallis post-test (three or more conditions). In all figures significance is coded as: *p < 0.05; **p < 0.01; ***p < 0.001.

### Electrophysiology and Data Analysis

Seven or eight days post transduction, organotypic cortical slices were transferred into a recording/perfusion chamber placed on the fixed-stage of an upright microscope (BX51WI, Olympus; fitted with 4× air and 40× water-immersion objectives, a Photonics VX55 camera and a BX-FLA illumination system) and superfused at 1–3 ml/min with artificial cerebrospinal fluid (ACSF) maintained at room temperature and saturated with 95% O_2_ and 5% CO_2_. Voltage-clamp recordings were performed with patch pipettes pulled from borosilicate glass (World Precision Instruments) and having an electrode resistance of 1.5–2.5 MΩ. Selective recording of Na^+^ currents was performed by using patch pipettes filled with an internal solution containing (in mM) 140 CsFl (to block K^+^ and hyperpolarization-activated cation currents), 1 MgCl_2_, 10 Hepes, 2 EGTA, 2 ATP, 0, 2 GTP (pH7, 4) and a modified ACSF solution (Na^+^ current isolation solution) containing (in mM): 120 NaCl, 3 KCl, 26 NaHCO_3_, 10 Glucose, 1 MgCl_2_. This external solution did not contain CaCl_2_ (to prevent Ca^2+^ currents) but was added with 10 mM tetraethyl-ammonium, 4 mM 4-Aminopyridine, 3 mM kynurenic acid (Sigma Aldrich) to attenuate K^+^ currents and glutamate synaptic transmission. Separate recording sessions were also performed with 500 nM TTX (Tetrodotoxin, Ascent Scientific) added to the Na^+^ current isolation solution in order to check that the recorded current was carried by Na^+^ ions. Na^+^ currents were evoked by voltage ramps reaching +40 mV from a holding potential of −90 mV and applied at a speed of 0.2 mV/ms.

All recordings were performed exclusively from GFP positive neurons using an Axopatch 200B (Molecular Devices). A computer interfaced to a 12-bit A/D converter (Digidata 1322A using Clampex 9.x; Molecular Devices LLC) controlled the voltage-clamp protocols and data acquisition. The signals were filtered at 5 KHz and digitized at 20 KHz. Uncompensated series resistance was 8 ± 0.5 MΩ. Analysis was conducted in Clampfit 10.3 (Molecular Devices) and SigmaPlot 12 (Systat Software Inc.). After off line linear leak subtraction, the following parameters were measured: threshold voltage for both Na^+^ persistent current (INaP) and Na^+^ transient currents (INaT), peak voltage for INaP and peak amplitudes for both INaP and INaT. Membrane potentials are uncorrected for the liquid junction potential (8.9 mV). Data are shown as mean ± SEM. Mann-Whitney rank sum test was used to test for statistical differences with a significance level of *p* < 0.05.

## Results

### Nav1 Knockdown Reduces Na^+^ Current in Organotypic Slices and Impairs AnkG Concentration

**Figure 1:**
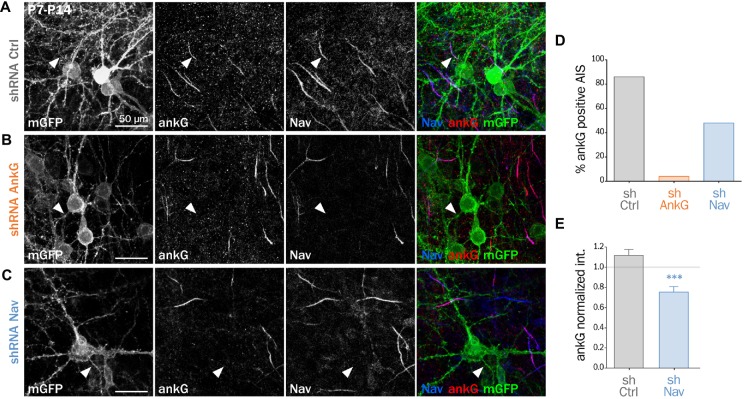
**Knockdown of Nav channels downregulates the axon initial segment (AIS) in organotypic slices.** Organotypic cortical slices prepared from postnatal day 7 rats were infected with a lentivirus co-expressing membranous GFP (mGFP) and shRNA directed against ankG (shAnkG), Nav (shNav) or luciferase as control (shCtrl). Seven days post-infection, slices were fixed, immunostained for GFP, ankG and Nav. **(A–C)** Maximum intensity projection of 15–20 optical slices. Scale bar: 50 μm. **(D)** Percentage of infected neurons with an observable AIS (shCtrl *n* = 80; shAnkG *n* = 27; shNav *n* = 97; sum of three independent experiments). **(E)** Ratio of the mean fluorescence intensity for ankG labeling at the AIS in transfected neurons (where the AIS was still detected) compared to the surrounding untransfected cells (shCtrl 1.10 ± 0.06, *n* = 69; shNav: 0.72 ± 0.05, *n* = 47; three independent experiments).

We and others have demonstrated that Nav1 clustering at the AIS requires a direct interaction between the ankyrin-binding-domain (ABD) of Nav channels and the MBD of ankG (Garrido et al., 2003; Gasser et al., 2012; Montersino et al., 2014). We wanted to know if the Nav-ankG interaction could conversely contribute to ankG concentration and AIS integrity. We first assessed the effect of Nav depletion on ankG concentration at the AIS in cultured organotypic slices (Stoppini et al., 1991). Cortical slices obtained from 7 day rats were transduced with a recombinant lentivirus expressing a previously validated shRNA directed against Nav1.1/Nav1.2/Nav1.3 (shNav; Xu and Shrager, 2005; Hedstrom et al., 2007; Hien et al., 2014) or against ankG (shAnkG; Hedstrom et al., 2007; Leterrier et al., 2011b). After 7 days, we evaluated the AIS integrity using immunostaining against Nav and ankG. The membrane-targeted GFP (mGFP) marker co-expressed with the shRNA allowed visualizing the full morphology of the transduced neurons (Figure 1). AnkG staining was readily observable in 86% of the neurons transduced with a control shRNA (Figures 1A,D) but was detected in only a small proportion of neurons expressing shAnkG (Figures 1B,D). In neurons transduced with shNav, ankG accumulation was observed in only 48% of the neurons (Figures 1C,D). As compared to control neurons, Nav1-depleted neurons had a significantly reduced ankG concentration at the AIS (ankG ratio—mean intensity of ankG labeling at the AIS and normalized to ankG labeling in surrounding non-infected neurons—down from 1.10 ± 0.06 in shCtrl neurons to 0.72 ± 0.05 in shNav neurons, Figure 1E). These results demonstrate that the depletion of Nav induces a significant decrease of ankG accumulation at the AIS.

**Figure 2:**
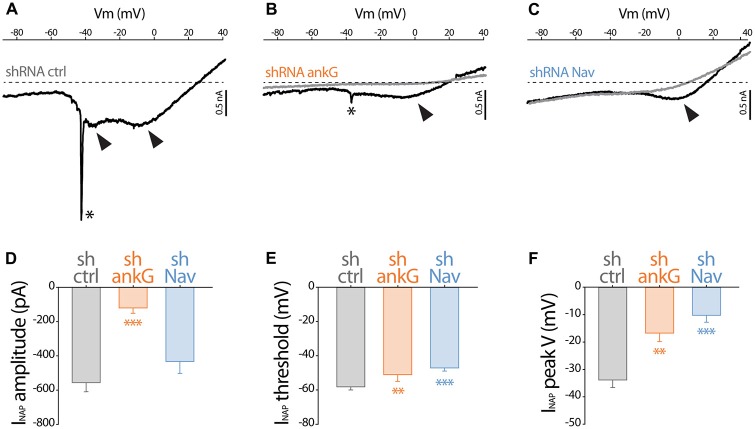
**Effect of ankG and Nav knockdown on sodium currents.** Patch-clamp recordings obtained from mGFP expressing neurons from organotypic cortical slices, 7–8 days post infection with shCtrl, shAnkG or shNav. **(A–C)** Raw traces of Na^+^ currents evoked by voltage ramps (from holding potential −90 mV to +40 mV at 0.2 mV/ms) in the absence (black) or presence (gray) of 500 nM TTX. The asterisks and arrows respectively indicate transient Na^+^ currents and components of the Na^+^ persistent current (INaP). **(D–F)** Characteristics of the INaP in the different conditions (shCtrl *n* = 19; shAnkG *n* = 5; shNav *n* = 9). **(D)** averaged peak amplitude; **(E)** peak threshold; **(F)** maximum peak voltage.

To evaluate how Na^+^ currents were affected by ankG or Nav silencing, we performed electrophysiological recording on cultured slices, 7–8 days after infection. In large neurons with fully developed processes, space clamp problems prevent adequate voltage clamp recording of rapidly inactivating, i.e., transient, Na^+^ current (INaT). However, all the Nav types expressed in central nervous system neurons generate also a slowly inactivating, i.e., persistent, Na^+^ current (INaP) that follows INaT (Mantegazza et al., 2005; Rush et al., 2005; Estacion and Waxman, 2013). Using depolarizing voltage ramps at adequate speed, we were able to record both correctly clamped INaP and unclamped INaT (Del Negro et al., 2002). The recorded currents were suppressed in the presence of TTX, showing that TTX-sensitive Nav channels generate these currents. In all the neurons transduced with the control shRNA, voltage ramps generated a large INaP that started to activate at −58.2 ± 1.8 mV (Figures 2A,D) and exhibited usually two components (Figure 2A), with the largest peaking at −33.9 ± 2.7 mV (Figure 2D). As expected, in most of these neurons (13/19), unclamped INaT were also generated and appeared as a single spike (star in Figure 2A; average threshold: −48.5 ± 1.4 mV; average peak amplitude: 2467 ± 648 pA). In neurons expressing shAnkG, INaP was affected both in terms of amplitude (strong reduction in 4/5 neurons and null current in 1/5 neuron; Figures 2B,D) and in terms of voltage dependence (depolarizing shift of the activation threshold and peak voltage; Figures 2E,F). In addition, in these ankG depleted neurons, INaT was in most cases absent (4/5 neurons) or extremely small (1/5 neurons, trace presented in Figure 2B; threshold: −39 mV; peak amplitude: 208 pA). In the neurons expressing shRNA against Nav, INaP exhibited significant changes in voltage dependence (more depolarized threshold and peak voltage, Figures 2E,F), but its average amplitude was not significantly lower than in shCtrl neurons (Figure 2D). Remarkably, INaT was absent in all the Nav-depleted neurons (9/9 neurons). Altogether these results confirm that Nav channels were functionally eliminated in our shRNA experiments.

### Nav or Nfasc186 Knockdown Impair AIS Maintenance in Cultured Neurons

**Figure 3:**
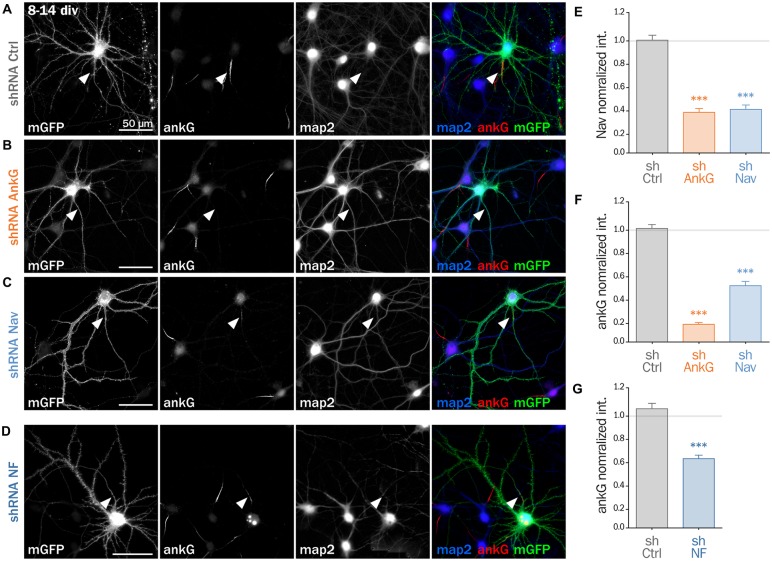
**Knockdown of AIS membrane components perturbs AIS maintenance.** Cultured rat hippocampal neurons transfected at 8 days *in vitro* (8 div) with mGFP and shCtrl **(A)**, shAnkG **(B)**, shNav **(C)** or shNF **(D)**, fixed 6 days later (14 div) and then immunostained for GFP, ankG, map2 and Nav. Arrowheads indicate the AIS of transfected neurons. **(E)** Ratio of the mean fluorescence intensity for Nav1 labeling at the AIS of transfected cells compared to surrounding untransfected cells (shCtrl 0.96 ± 0.04, *n* = 43; shAnkG 0.35 ± 0.03, *n* = 43; shNav 0.37 ± 0.04, *n* = 40; three independent experiments). **(F,G)** Ratio of the mean fluorescence intensity for ankG labeling at the AIS in transfected neurons compared to surrounding untransfected cells (**F**: shCtrl 0.97 ± 0.03, *n* = 116; shAnkG *n* = 0.15 ± 0.01, *n* = 94; shNav 0.48 ± 0.04, *n* = 123; six independent experiments. **G**: shCtrl 1.02 ± 0.05, *n* = 36; shNF 0.59 ± 0.03, *n* = 64; three independent experiments).

To decipher the mechanisms involved in the interplay between Nav and ankG, we tested the effect of Nav depletion in cultured hippocampal neurons, an amenable model for the analysis of neuronal morphology and polarity (Kaech and Banker, 2006). Neurons were maintained for 8 div, ensuring that polarity was well established, then transfected with shRNA constructs, and fixed 6 days later (14 div; Figure 3). We first verified that the ankG labeling intensity was strongly reduced in shAnkG transfected neurons, compared to untransfected surrounding neurons (Figure 3B): ankG intensity ratio between transfected and untransfected neurons was 0.97 ± 0.03 for shCtrl, but only 0.15 ± 0.01 for shAnkG (Figure 3F). Similarly, we checked the efficiency of Nav depletion and found that the Nav intensity ratio was 0.96 ± 0.04 for shCtrl and 0.37 ± 0.04 for shNav (Figure 3E). As expected, AnkG depletion also resulted in Nav disappearance (Nav ratio 0.35 ± 0.03, Figure 3E). Consistently with our results in slices, Nav1 depletion was accompanied by a ~50% decrease in ankG labeling in the AIS (ankG ratio 0.48 ± 0.04, Figure 3F), but did not affect the position and length of the AIS that remained unchanged. Thus, Nav1 expression is required for ankG concentration at the AIS, and therefore for AIS maintenance. We reasoned that Nav could have a stabilizing effect by anchoring ankG to the membrane. Since the MBD of ankG can bind both to Nav and CAMs such as Nfasc186, we examined whether Nfasc186 can also participate in ankG stabilization at the AIS, as suggested by data obtained after conditional genetic depletion (Zonta et al., 2011). Six days after transfection of 8 div neurons with a validated shRNA against Nfasc186 (Hedstrom et al., 2007), ankG accumulation at the AIS was quantitatively analyzed. AnkG labeling was significantly reduced in neurons depleted for Nfasc186 (1.02 ± 0.05 for shCtrl, 0.59 ± 0.03 for shNF, Figures 3D,G). This additional observation suggests that elimination of membrane partners of ankG (Nav or CAMs) is sufficient to induce AIS destabilization in polarized neurons.

### Nav or Nfasc186 Knockdown Impairs AIS Formation

**Figure 4:**
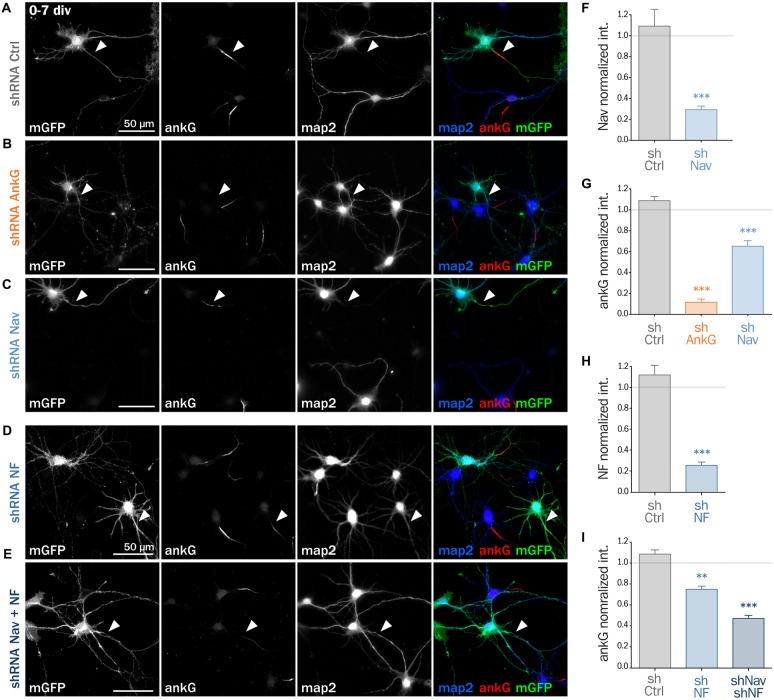
**Knockdown of AIS membrane components impairs AIS formation.** Rat hippocampal neurons were transfected before plating (0 div) with mGFP and shCtrl **(A)**, shAnkG **(B)**, shNav **(C)**, shNF **(D)** or shNav + shNF **(E)**. Seven days later (7 div), neurons were fixed and immunostained for GFP, ankG, map2 and Nav or Nfasc186. Arrowheads indicate the AIS of transfected neurons. **(F)** Ratio of the mean fluorescence intensity for Nav1 labeling at the AIS of transfected neurons compared to surrounding untransfected neurons (shCtrl 1.08 ± 0.12, *n* = 41; shNav 0.30 ± 0.03, *n* = 55; three independent experiments). **(H)** Ratio of the mean fluorescence intensity for Nfasc186 labeling at the AIS of transfected neurons compared to surrounding untransfected neurons (shCtrl 1.12 ± 0.09, *n* = 20; shNF 0.25 ± 0.03, *n* = 25; two independent experiments). **(G,I)** Ratio of the mean fluorescence intensity for ankG labeling at the AIS of transfected neurons compared to surrounding untransfected neurons (**G**: shCtrl 1.08 ± 0.04, *n* = 80; shAnkG 0.12 ± 0.03, *n* = 40; shNav 0.65 ± 0.05, *n* = 39; three independent experiments. **I**: shCtrl 1.08 ± 0.04, *n* = 80; shNF 0.75 ± 0.03, *n* = 43 shNav + shNF 0.47 ± 0.03, *n* = 109; three independent experiments).

Next, we assessed if ankG membrane partners are also required for proper AIS formation in young neurons. In developing hippocampal neurons in culture, polarity is established around 2 div and is quickly followed by AIS assembly (Dotti et al., 1988; Hedstrom et al., 2007). Freshly dissociated hippocampal neurons were thus nucleofected in order to express shRNA prior to AIS formation and then fixed after 7 div (Figures 4A–E). We first checked that the expression of each shRNA led to the specific down regulation of its corresponding targets: ankG (ankG ratio 1.08 ± 0.04 for shCtrl; 0.12 ± 0.03 for shAnkG, Figure 4G), Nav1 (Nav ratio 1.08 ± 0.12 for shCtrl; 0.30 ± 0.03 for shNav, Figure 4F) or Nfasc186 (Nfasc186 ratio 1.12 ± 0.09 for shCtrl, 0.26 ± 0.03 for shNF, Figure 4H). As observed in mature neurons, ankG concentration was impaired in neurons depleted for either Nav channels (ankG ratio 0.65 ± 0.05 for shNav, Figure 4G) or Nfasc186 (ankG ratio 0.75 ± 0.03 for shNF, Figure 4I). In addition, we found that the effects of Nav or Nfasc186 depletion on AIS formation were cumulative, as simultaneous depletions using both shRNAs (shNav + shNF) further reduced ankG concentration at the AIS (0.47 ± 0.03, Figure 4I). Altogether, these data demonstrate that the membrane partners of ankG, Nav and Nfasc186 are essential for AIS assembly.

### Nav1.6-GFP Expression Rescues the AnkG Downregulation Induced by Depleting Nav or Nfasc186

**Figure 5:**
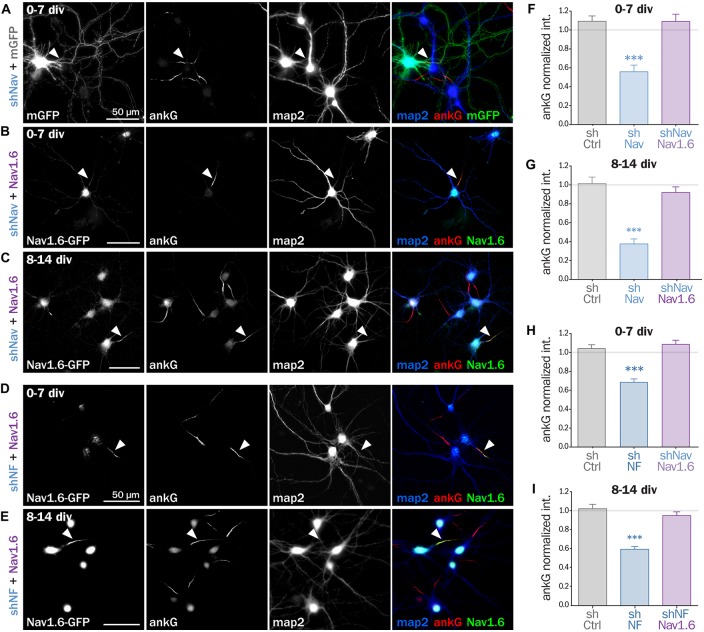
**Nav1.6 rescue the AIS downregulation induced by Nav knockdown, but also by Nfasc186 knockdown.** Rat hippocampal neurons co-transfected at 0 div or 8 div with mGFP or Nav1.6-GFP and shNav **(A–C)** or shNF **(D,E)** and fixed 6 or 7 days later (0–7 div or 8–14 div) for subsequent immunostaining for GFP, ankG and map2. Arrowheads indicate the AIS of transfected neurons. **(F–I)** Normalized mean fluorescence intensity for ankG labeling at the AIS of 0–7 div **(F,H)** or 8–14 div **(G,I)** transfected neurons. (**F**: shCtrl 1.09 ± 0.06, *n* = 54; shNav 0.56 ± 0.07, *n* = 21, shNav + Nav1.6 1.09 ± 0.07, *n* = 41; three independent experiments. **G**: shCtrl 1.01 ± 0.07, *n* = 28; shNav 0.37 ± 0.05, *n* = 39; shNav + Nav1.6 0.92 ± 0.06, *n* = 30; two independent experiments. **H**: shCtrl 1.04 ± 0.04, *n* = 61; shNF 0.68 ± 0.04, *n* = 64; shNF + Nav1.6 1.09 ± 0.04, *n* = 68; three independent experiments. **I**: shCtrl 1.02 ± 0.05, *n* = 36; shNF 0.59 ± 0.03, *n* = 64; shNF + Nav1.6 0.95 ± 0.04, *n* = 58; three independent experiments).

If Nav channels specifically contribute to AIS formation and integrity, ankG downregulation induced by Nav depletion should be rescued by the co-expression of an shRNA-resistant Nav construct. To test this hypothesis, we used a full length Nav1.6-GFP that is resistant to our shRNA construct against Nav (three bases mismatch; Gasser et al., 2012). Nav1.6-GFP and a shNav plasmid containing soluble Td-Tomato as transfection marker were co-expressed either in freshly dissociated or mature (8 div) hippocampal neurons that were analyzed 6–7 days later. By contrast with mGFP that filled the entire neuron, Nav1.6-GFP was concentrated in the soma and at the AIS of co-transfected cells (Figures 5A–C). Notably, over-expression of Nav1.6-GFP alone did not alter the AIS morphology or ankG content (not shown). Quantitative analysis showed that ankG concentration was restored at the AIS by Nav1.6-GFP co-expression either in young neurons (0.56 ± 0.07 for shNav + mGFP, 1.09 ± 0.08 for shNav + Nav1.6-GFP, Figure 5F) or in mature neurons (0.37 ± 0.05 for shNav + mGFP, 0.92 ± 0.06 for shNav + Nav1.6-GFP, Figure 5G). These data validate the specificity of the effects observed with the shNav.

If ankG stabilization depends on ankG membrane anchoring *per se* and not on the partner identity, the overexpression of Nav1.6-GFP should also be able to cross-rescue Nf186 depletion (Figures 5D,E). When Nav1.6-GFP was co-expressed with the shRNA against Nfasc186, we indeed observed that ankG concentration at the AIS was restored in both young (0.68 ± 0.03 for shNF + mGFP, 1.09 ± 0.04 for shNF + Nav1.6-GFP, Figure 5H) and mature neurons (0.59 ± 0.03 for shNF + mGFP, 0.95 ± 0.04 for shNF + Nav1.6-GFP Figure 5I). This cross-rescue experiment demonstrates that one ankG membrane partner can be replaced by another and that the identity of the partner is less important than the membrane anchoring itself to drive AIS formation and maintenance.

### Expression of a Chimeric Membrane-Anchored AnkG Partner Rescues AIS Formation

**Figure 6:**
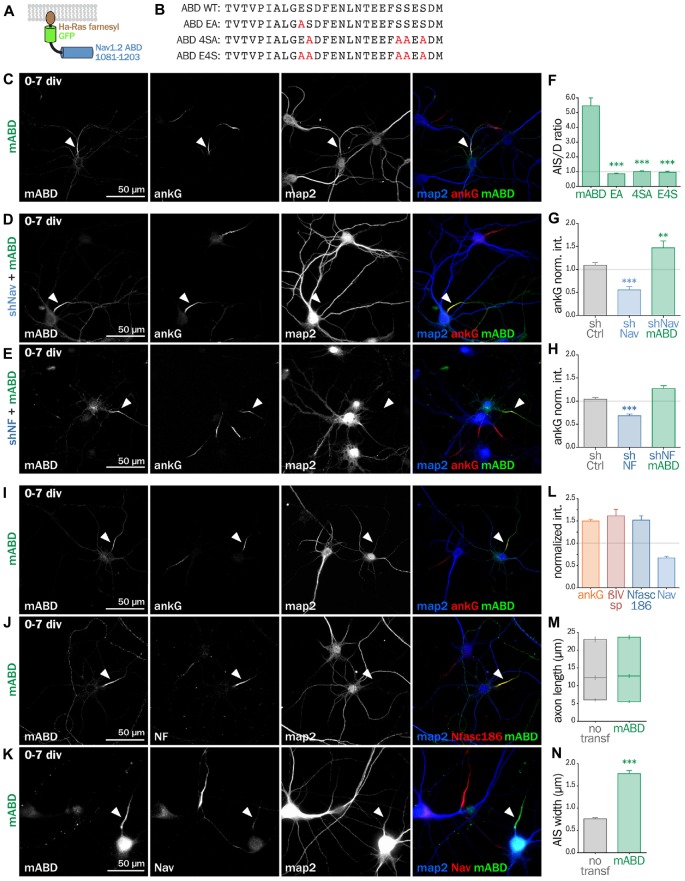
**Synthetic mABD construct rescues Nav or Nfasc186 knockdown and upregulates AIS formation. (A)** Schematic representation of the mABD construct composed of the amino-acids 1081–1203 of rat Nav1.2 fused to a GFP with a farnesylation motif from Ha-Ras in its C-terminal. **(B)** Alignment of wild type (WT) and mutated sequences corresponding to the rat Nav1.2 (aa 1102–1128) Ankyrin Binding Domain (ABD). The mutated amino-acids involved in ankG binding are in red. Rat hippocampal neurons transfected at 0 div with mABD alone **(C,I,J,K)** or in association with shNav **(D)** or shNF **(E)**, fixed 7 days later (div 7), and immunostained for GFP, map2 and ankG **(C–E,I)**, Nfasc186 **(J)** or Nav **(K)**. Arrowheads indicate the AIS of transfected neurons. **(F)** Ratio of the mean fluorescence intensity for GFP labeling in the proximal axon compared to the mean of three dendrites **(D)** in each transfected neuron (mABD 5.46 ± 0.54, *n* = 77, mABD-EA 0.86 ± 0.06, *n* = 37, mABD-4SA 1.01 ± 0.05, *n* = 36; mABD-E4S 0.96 ± 0.07, *n* = 32; two independent experiments). **(G,H)** Ratio of the mean fluorescence intensity for ankG labeling at the AIS of transfected neurons compared to surrounding untransfected neurons (**G**: shCtrl 1.09 ± 0.06, *n* = 54; shNav 0.56 ± 0.07, *n* = 21; shNav + mABD 1.47 ± 0.15, *n* = 23; two independent experiments. **H**: shCtrl 1.04 ± 0.04, *n* = 61; shNF 0.68 ± 0.04; *n* = 64; shNF + mABD 1.27 ± 0.07, *n* = 49; two independent experiments). **(L)** Ratio of the mean fluorescence intensity for ankG, ßIV-spectrin (ßIVsp), Nfasc186 or Nav labeling at the AIS of transfected neurons compared to surroundings untransfected neurons (ankG 1.50 ± 0.04, *n* = 146; ßIVsp 1, 52 ± 0.10, *n* = 49; NF 1, 62 ± 0.14, *n* = 26; Nav0.67 ± 0.04; *n* = 33; 2–6 independent experiments). **(M,N)** Length and width of the AIS in mABD-transfected neurons (untransfected cells AIS width 0.76 ± 0.03 μm, *n* = 61; mABD 1.78 ± 0.07 μm, *n* = 60; two independent experiments).

Next, we wanted to directly prove that ankG association to the membrane *via* a protein partner contributes to ankG targeting and assembly. We devised a minimal chimeric protein bearing the well-described ABD from the intracellular loop II-III of Nav1.2 (Garrido et al., 2003; Lemaillet et al., 2003; Gasser et al., 2012) fused to the farnesylated form of GFP that associates to the plasma membrane (Figures 6A,B). When this membrane ABD (mABD) construct was expressed in freshly dissociated hippocampal neurons, it was highly concentrated along the proximal axon with an AIS/dendrite ratio of 5.46 ± 0.53 (Figures 6C,F). As ABD binding to ankG is known to be impaired by the mutation of glutamate residue Nav1.2 E1111 or the four serine residues implicated in CK2 regulation (Bréchet et al., 2008), we produced three mutant constructs where E1111, the four serine residues or all these five amino-acids were replaced by alanine (mABD-EA, mABD-4SA and mABD-E4S; Figure 6B). These mutations abolished the ability of mABD to be concentrated to the AIS, resulting in an AIS/dendrite ratio close to 1 (Figure 6F). This indicates that mABD concentration at the AIS is under the control of a phospho-dependent interaction with ankG. We next assessed whether the expression of mABD was able to rescue the ankG downregulation induced by either Nav or Nf186 depletion. In young neurons, mABD expression rescued the deficit in ankG accumulation caused by depletion of either Nav (ankG ratio 1.47 ± 0.15 for shNav + mABD; Figures 6D,F,G) or Nfasc186 (ankG ratio 1.27 ± 0.07 for shNF + mABD, Figures 6E,H). Notably, we observed that the over-expression of mABD by itself up-regulated AIS components such as ankG, ß4-spectrin and Nfasc186 (ratios of 1.50 ± 0.04 for ankG, 1.52 ± 0.09 for ß4 spectrin, and 1.62 ± 0.14 for NF, Figures 6I,J,L). By opposition, endogenous Nav were strongly downregulated, indicating that the ABD domain acts as a dominant negative on endogenous Nav targeting or anchoring during AIS formation (Nav ratio 0.67 ± 0.04, Figures 6K,L). In addition, mABD expression affected the morphology of the AISs that were wider, but not longer, than those of untransfected neurons (length 17.7 ± 0.8 μm for untransfected, 18.4 ± 0.7 μm for mABD, width 0.76 ± 0.02 μm for untransfected, 1.78 ± 0.07 μm for mABD, Figures 6M,N). Altogether, these experiments performed during AIS formation show that mABD is able to rescue the absence of Nav or Nfasc186, and that its overexpression upregulates the whole AIS assembly, leading to a wider AIS that accumulates a higher density of components at the exception of Nav channels.

### mABD Expression in Mature Neurons Results in AnkG Ectopic Localization

**Figure 7:**
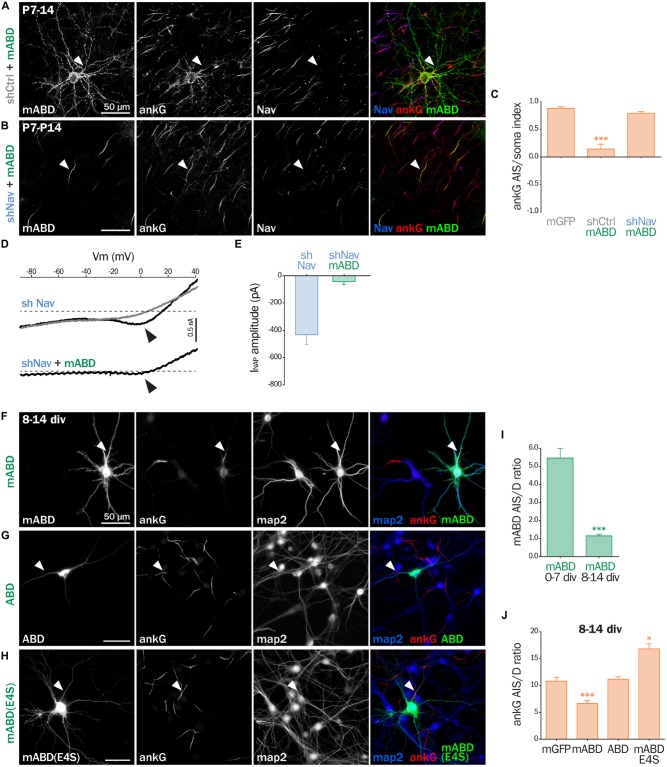
**mABD expression in mature neurons results in ankG mislocalization. (A–E)** Organotypic cortical slices prepared from postnatal day 7 rats infected with a lentivirus co-expressing mABD and shCtrl or shNav and either treated for immunostaining for GFP, ankG and Nav **(A–C)** or used for patch-clamp recording **(D,E)** 7–8 days post-infection. **(A,B)** Maximum-intensity projection of 15–20 optical slices: arrowheads indicate the AIS of transfected neurons. **(C)** Polarity index of ankG labeling in the AIS vs. the soma obtained from infected neurons (mGFP alone 0.88 ± 0.03, *n* = 8; mABD + shCtrl 0.15 ± 0.09, *n* = 10; mABD + shNav 0.79 ± 0.03, *n* = 10; two independent experiments). **(D)** Raw traces of INaP evoked by voltage ramps in the absence (black) or presence (gray) of 500 nM TTX. Arrowheads indicate the INaP. **(E)** Averaged INaP peak amplitude (shNav *n* = 9; shNav + mABD: *n* = 3/7). **(F–J)** Rat hippocampal neurons transfected at 8 div with mGFP, mABD, ABD (mABD devoid of its membrane anchor) or mABD-E4S (mABD invalidated for ankG interaction, see Figure 6B) fixed 6 days later (8–14 div) and immunostained for GFP, map2 and ankG. Arrowheads indicate the AIS of transfected neurons. **(I)** Ratio of the mean fluorescence intensity for GFP labeling in the proximal axon compared to the mean intensity of three dendrites in each transfected neuron (mABD expressed from 0 div to 7 div from Figure 6F for comparison, mABD expressed from 8 div to 14 div 1.15 ± 0.09, *n* = 66, two independent experiments). **(J)** Ratio of the mean fluorescence intensity for ankG labeling in the proximal axon compared to the mean intensity of three dendrites in each transfected neuron (mGFP 10.79 ± 0.73, *n* = 30; mABD 6.64 ± 0.57, *n* = 114; ABD 11.16 ± 0.51, *n* = 58; mABD-E4S 16.83 ± 0.95, *n* = 66; 2–4 independent experiments).

Next, we assessed the effect of mABD expression in mature neurons. First, we turned back to post-natal cortical organotypic slices, and examined neurons transduced with mABD (together with a control shRNA). In these mature neurons with an already assembled AIS, mABD was localized in the whole neuron, with no concentration at the AIS (Figure 7A). This contrasted with the AIS localization observed when expressing mABD during AIS formation in cultured neurons (see above). Interestingly, this non-polarized expression of mABD was accompanied by a partial delocalization of ankG, which was found to accumulate in the soma and proximal dendrites in addition to the AIS (Figure 7A). This was evidenced by the drop of the AIS/soma polarity index for ankG labeling in neurons expressing mABD compared to neurons expressing the neutral marker mGFP (0.88 ± 0.03 for mGFP, 0.14 ± 0.09 for mABD, Figure 7C). We reasoned that this difference in mABD localization observed during AIS formation compared to the already assembled AIS could be explained by the presence, in the AIS of mature neurons, of strongly anchored endogenous Nav that compete with the mABD construct for ankG binding sites. To test this hypothesis, the mABD together with the shRNA against Nav channels were expressed in organotypic slices. In contrast to control neurons, in the Nav-depleted neurons, the mABD was concentrated to the AIS and did not delocalize ankG (Figures 7B,C; ankG polarity index for mABD + shNav: 0.79 ± 0.03). Thus, the expression of mABD in mature neurons depleted for Nav results in a proper stabilization of ankG at the AIS. This also means that mABD expression in organotypic slices rescues the ankG downregulation observed in neurons depleted for Nav (see Figure 1). To functionally confirm that Nav depletion did occur in these neurons having a morphologically normal AIS, we performed electrophysiological experiments on neurons co-expressing shNav and mABD (Figures 7D,E). In these neurons, no INaT could be recorded (7/7 neurons). More remarkably, INaP was either absent (4/7 neurons) or extremely small with an average amplitude as small as 42, 3 ± 21, 5 pA (3/7 neurons; Figures 7D,E). In the seven slices from which these mABD positive neurons were patched, we also recorded Na^+^ currents from non-transfected neurons in which the electrophysiological parameters of both INaT and INaP were similar to the currents recorded in control neurons as shown in Figure 2. These results demonstrate that a morphologically normal AIS can be maintained in neurons devoid of Na^+^ currents. To confirm the ectopic localization of ankG observed in organotypic slices, we assessed the effect of overexpressing mABD in cultured mature hippocampal neurons (Figures 7F–J). In these neurons, mABD was also localized in the whole neuron with no preferential accumulation at the AIS (AIS/dendrite ratio for mABD 1.15 ± 0.09, Figures 7F,I). mABD over-expression also produced a mislocalization of ankG, that appeared in the cell body and proximal dendrites in addition to the AIS (AIS/dendrite ratio from 10.79 ± 0.73 in control neurons to 6.64 ± 0.57 in mABD-expressing neurons, Figures 7F,J). We determined if the two properties of the mABD construct (membrane association and ankG binding) were necessary for this delocalization of ankG. The ABD construct devoid of the farnesylation motif was expressed in a non-polarized manner, and was not able to mislocalize ankG (Figures 7G,J). Similarly, the mABD-E4S mutant that lacks ankG binding was localized in a non-polarized manner, and did not delocalize ankG from the AIS (Figures 7H,J). Overall, the expression of mABD in mature neurons from slices and cultures shows that binding of ankG to its membrane partners is important for its stabilization at the AIS. Furthermore, the ectopic localization of ankG after mABD expression suggests that ankG binding by mABD is sufficiently strong to perturb ankG targeting and that the association of ankG with its membrane partners occurs upstream of their insertion into the AIS scaffold.

## Discussion

The AIS is a highly specialized neuronal compartment that plays a key role in neuronal development and excitability. Many studies have highlighted the central role of ankG in the establishment and maintenance of the AIS molecular scaffold, since it targets and anchors most, if not all, known AIS components. Such central role in AIS building and stabilization have been demonstrated by Hedstrom et al. (2007) that obtained disappearance of the AIS only with ankG knockdown, as we show in Figure 3B. However, the converse role of the AIS components on ankG trafficking and stabilization is still poorly understood. Although many studies have reported that the down-regulation of non-membranous AIS proteins drives no or weak ankG concentration decrease (Leterrier et al., 2011b; Hien et al., 2014; Papandréou et al., 2015; Fréal et al., 2016; Pablo et al., 2016), we have demonstrated here that Nav and Nfasc186 knockdown in both developing and mature neurons induced a significant and cumulative decrease of ankG. This suggests that the membranous partners of ankG play a cardinal role in the interactome constituted by the AIS resident proteins. Thus, although ankG is unquestionably necessary for AIS construction and stabilization, it is not sufficient by itself to ensure the complete assembly of the AIS.

Both Nav and Nfasc186 are membrane proteins that bind directly to the MBD of ankG *via* their ABD. Their individual knockdown produced a similar decrease of ankG concentration at the AIS, whereas their simultaneous depletion had a cumulative effect (although their ABD are different). The very low transfection rate in our neuronal cultures prevented to determine whether this decrease of ankG concentration at the AIS reflects loss of expression, degradation or mislocalization. Nevertheless, this significant down-regulation indicates that the ability of Nav and Nfasc186 to anchor ankG to the plasma membrane plays a role in ankG targeting and maintenance at the AIS. Furthermore, the ankG down-regulation induced by Nfasc186 depletion is rescued by expressing recombinant Nav channels, which demonstrates that, regardless the identity of the anchoring membrane partner, membrane association is a crucial step in ankG targeting. Such a role of the association between ankG and Nav or Nfasc186 is consistent with recent data from Wang et al. (2014) who resolved the MBD structure. Indeed, mutations in two sites of the MBD that mediate binding to Nfasc186 also prevent the correct targeting of recombinant ankG to the AIS of cultured hippocampal neurons. Interestingly, a direct ankG association with the plasma membrane was also showed to be necessary by He et al. (2012). These authors observed that the suppression of the cysteine 70 of ankG, which is required for its S-palmitoylation, led to a loss of ankG accumulation to the AIS. We have not tested whether other AIS membrane partners contribute to ankG targeting. Nevertheless, we can suspect that KCNQ2/3 (Kv7.2/Kv7.3) channels, which have an ABD very similar to that of Nav channels, could have a similar stabilizing effect (Pan et al., 2006; Hill et al., 2008; Xu and Cooper, 2015).

To pinpoint the features required for ankG stabilization by its membrane partners, we designed a minimal chimeric protein consisting in a membrane-anchored ankyrin binding domain (mABD). This chimeric protein had a dominant negative effect on the concentration of endogenous Nav channels at the AIS. This effect was confirmed by our electrophysiological data obtained in cultured organotypic cortex slices. In this model, neurons expressing shNav alone exhibited a residual INaP (likely produced by Nav1.6 that is not targeted by our shRNA) that was suppressed in neurons co-expressing shNav and mABD. Although mABD expression had a dominant negative effect on endogenous Nav, it rescued the ankG downregulation induced by the shNav and drove the reestablishment of an AIS morphologically indistinguishable from the AIS of naive neurons (see Figure 7B). This demonstrates that a properly assembled AIS can be reconstituted by mABD in neurons devoid of Na^+^ currents. Thus, the membrane-anchoring effect of Nav channels is essential for ankG targeting and anchoring to the AIS, independently of their ability to produce Na^+^ currents.

Our study unravels two mechanisms regarding AIS components assembly and maintenance. First, we evidenced an unexpected ability of the AIS structure to be plastic in young neurons. Indeed, when mABD is overexpressed during AIS formation, a larger amount of ankG is positioned at the proximal axon, promoting a wider AIS. These observations suggest that the diameter of the AIS is subjected to a modulation regulated by the amount of available membrane components. These data fit with the observation that the level of Nav channels expressed to the membrane is regulated by a combination of mechanisms involving numerous Nav interacting partners such as ankG, CK2, VGSC beta subunits, FGF13- and 14 (Hien et al., 2014; Montersino et al., 2014; O’Malley and Isom, 2015; Pablo et al., 2016). This AIS diameter change could influence electrogenesis properties, as shown for AIS length and position plasticity in developing neurons (Galiano et al., 2012; Gutzmann et al., 2014; Kuba et al., 2014). On the opposite, in mature neurons the assembled AIS exhibit very little plasticity unless they are destabilized. Indeed, overexpression of mABD in mature cortical neurons leads to an ectopic localization of ankG that is rescued by a concomitant knockdown for Nav channels. Our data are consistent with the observed stability of AIS components in mature neurons (Hedstrom et al., 2008; Akin et al., 2015) and the preferential regulation of channels immobilization by CK2 in young neurons (Brachet et al., 2010). A second important finding is the fact that ankG could interact with its membrane partners outside the AIS. Indeed, the ability of mABD to mistarget ankG in mature neurons suggests that ankG and its membrane partners, in particular Nav channels, can physically interact with each other upstream of their insertion into the AIS scaffold, and are likely co-transported to the AIS. The mechanisms for the targeting of AIS proteins are the subject of an ongoing debate. On the one hand, a “diffusion trapping” model has been proposed in which Nav and KCNQ2/3 channels are transported to the plasma membrane and diffuse to the AIS where they are immobilized by ankG (Leterrier et al., 2011a; Xu and Cooper, 2015). On the other hand, a “direct insertion” model was put forward in which Nav channels are directly targeted to the AIS where they are immediately immobilized (Barry et al., 2014; Akin et al., 2015). Our model of a co-transport of ankG associated to its membrane partners is in line with the latter hypothesis. We propose that ankG is targeted to vesicular membranes by a tight association with its membranous partners. This implies that the ankG/membrane partner complexes are co-transported to the axon, presumably via kinesin-1 (Barry et al., 2014), and inserted into the AIS scaffold during AIS formation and maintenance.

## Author Contributions

CL, NC and FC: conception and design; experiments and data acquisition; analysis and interpretation of data; draft and revision of the article. FR-B: experiments and data acquisition. AM: experiments and data acquisition; analysis and interpretation of data. BD: conception and design; draft and revision of the article.

## Funding

This work was supported by the Centre National pour la Recherche Scientifique, by grants to BD from the French Agence Nationale de la Recherche (ANR-2011-BSV4-001-1) and from Conseil Regional PACA (N°2011-10925).

## Conflict of Interest Statement

The authors declare that the research was conducted in the absence of any commercial or financial relationships that could be construed as a potential conflict of interest. The reviewer DM and handling Editor declared their shared affiliation, and the handling Editor states that the process nevertheless met the standards of a fair and objective review.

## Footnotes

^1^http://rsb.info.nih.gov/ij/

^2^https://github.com/cleterrier/ProFeatFit
